# Acoustic behavior of humpback whale calves on the feeding ground: Comparisons across age and implications for vocal development

**DOI:** 10.1371/journal.pone.0303741

**Published:** 2024-05-29

**Authors:** Julia M. Zeh, Dana L. Adcock, Valeria Perez-Marrufo, Dana A. Cusano, Jooke Robbins, Jennifer E. Tackaberry, Frants H. Jensen, Mason Weinrich, Ari S. Friedlaender, David N. Wiley, Susan E. Parks

**Affiliations:** 1 Department of Biology, Syracuse University, Syracuse, New York, United States of America; 2 Center for Coastal Studies, Provincetown, Massachusetts, United States of America; 3 Department of Ecoscience, Aarhus University, Roskilde, Denmark; 4 Biology Department, Woods Hole Oceanographic Institution, Woods Hole, Massachusetts, United States of America; 5 Whale Center of New England, Gloucester, Massachusetts, United States of America; 6 Institute of Marine Sciences, University of California Santa Cruz, Santa Cruz, California, United States of America; 7 Stellwagen Bank National Marine Sanctuary, Scituate, Massachusetts, United States of America; McGill University, CANADA

## Abstract

Studying sound production at different developmental stages can provide insight into the processes involved in vocal ontogeny. Humpback whales (*Megaptera novaeangliae)* are a known vocal learning species, but their vocal development is poorly understood. While studies of humpback whale calves in the early stages of their lives on the breeding grounds and migration routes exist, little is known about the behavior of these immature, dependent animals by the time they reach the feeding grounds. In this study, we used data from groups of North Atlantic humpback whales in the Gulf of Maine in which all members were simultaneously carrying acoustic recording tags attached with suction cups. This allowed for assignment of likely caller identity using the relative received levels of calls across tags. We analyzed data from 3 calves and 13 adults. There were high levels of call rate variation among these individuals and the results represent preliminary descriptions of calf behavior. Our analysis suggests that, in contrast to the breeding grounds or on migration, calves are no longer acoustically cryptic by the time they reach their feeding ground. Calves and adults both produce calls in bouts, but there may be some differences in bout parameters like inter-call intervals and bout durations. Calves were able to produce most of the adult vocal repertoire but used different call types in different proportions. Finally, we found evidence of immature call types in calves, akin to protosyllables used in babbling in other mammals, including humans. Overall, the sound production of humpback whale calves on the feeding grounds appears to be already similar to that of adults, but with differences in line with ontogenetic changes observed in other vocal learning species.

## Introduction

Studying individual variation in sound production across different development stages can provide insight into vocal ontogeny across species. Vocal ontogeny is a combination of changes in sound production related to physical maturation as well as changes in response to social feedback. As an animal grows, multiple components of their vocal repertoire change in relation to morphological changes in their vocal production organs. For example, call frequency often decreases with increasing body size throughout development, in line with the source-filter theory of vocal production (e.g., [1–4]). Changing vocal tract morphology may also allow for a greater acoustic space for vocalizations and a larger vocal repertoire, which is known as the expansion stage in humans but also occurs in other mammals [5–7]. Additionally, physical maturity may facilitate call refinement, leading to less frequency modulation, disorder, and noise [4, 8, 9].

Vocal learning is a multidimensional trait that includes varying degrees of both vocal usage learning (learning the context and timing for call use) and vocal production learning (production of modified or novel calls based on experience) [10–12]. One example of vocal usage learning is in the rules of vocal exchanges—like call timing, matching, and turn-taking—which are learned during ontogeny in some bird and mammal species, including human and non-human primates (e.g., [13–15]). For animals that produce bouts of vocalizations, like birdsong, the timing at which calls are produced relative to other calls in the same bout (i.e., the sequence’s rhythm) may be learned and developed over time [16].

Vocal production learning occurs across different timescales in different species. Much of the foundational work on the ontogeny of vocal production learning comes from studies of birdsong (e.g., [17, 18]) and human language development (e.g., [5]). In many songbirds, there is a sensitive period early in life for song learning that includes a sensory phase, when individuals hear model songs and create an auditory template, and a sensorimotor phase, when individuals figure out the motor program for vocal production according to their auditory template [17–19]. Within this sensorimotor phase, songbirds will produce subsong first, followed by plastic song, and finally crystallized, or mature adult, song [17, 18]. Young zebra finches (*Taeniopygia guttata*) often produce similar sounds in sequences that become more diverse as they age, and they also tend to sing syllable “prototypes” [19]. Humans also follow a sensory and sensorimotor phase of learning where the sensorimotor phase consists of plastic vocal production, often babbling, followed eventually by mature speech [5]. The sensitive phase of vocal learning varies across taxa, with some species labeled closed-ended learners that only learn during a short period early in development [20] and others that are open-ended learners that continue to refine vocal production and learn new sounds throughout their lives [17, 18, 21].

Compared to birds, vocal production learning appears to be relatively rare and is not as well understood in non-human mammals [10, 22]. It is especially challenging to tease apart the role of vocal tract maturation and learning in ontogeny–as well as to differentiate the influence of usage learning versus production learning–using observational data, since longitudinal or experimental lab studies are often lacking and infeasible. Call refinement and repertoire expansion have been observed in many mammals, but the underlying mechanism is still unclear. For example, juvenile sperm whales (*Physeter macrocephalus)* have a more diverse acoustic repertoire than adults and it takes some time before juveniles begin to refine their vocal behavior and use the repertoire specific to their social group [23, 24]. In elephant seals (*Mirounga leonine* and *Mirounga angustirostris*), vocalizations progressed from non-structured and variable to more stereotyped and structured over development [4, 25]. As mentioned before, repertoire expansion and call refinement with age could result from maturational changes, learning, or some combination of the two. Some bat species (one of the few taxa where longitudinal and experimental data exist) follow a similar ontogenetic trajectory in vocal production learning to humans and birds [6, 26]. This includes babbling, which is part of sensorimotor learning and practice.

Babbling is defined by adult-like sounds and immature vocalizations known as protophones in humans or protosyllables more broadly [5, 6, 27, 28]. Babbling behavior often occurs without social context and is repeated in sequences, sometimes with rhythmic structure (e.g., [5, 6, 28–30]). Human infants will use repetition and speech-like vocalizations as a form of vocal production practice and exploration [5, 27, 28, 31, 32]. In addition to humans and songbirds, babbling has also been observed in some species of bats [6, 30] and non-human primates [29], with some evidence of possible babbling also existing for bottlenose dolphins (*Tursiops truncatus;* [33]) and giant otters (*Pteronura brasiliensis;* [34]).

Humpback whales (*Megaptera novaeangliae*) are one of the species of mammals with evidence of vocal production learning [22]. This cosmopolitan species migrates annually between low-latitude breeding grounds and mid- to high-latitude feeding grounds [35]. Humpback whales rely on acoustic signals to communicate in a variety of contexts, and their complex vocal repertoire includes both song, which has been recorded only from males predominantly on the breeding grounds, and non-song social calls, which have been recorded across diverse individuals and contexts [36]. Evidence of song learning comes from studies of cultural transmission of novel song types over time and space [22, 37, 38]. As a vocal learning mammal, understanding vocal ontogeny in humpback whales is valuable from a comparative perspective relative to birds and other mammalian taxa across the vocal learning continuum.

Although the repertoire of mature adults is well studied, less is known about the vocal behavior of immature individuals. Humpback whales are born on low-latitude breeding grounds in winter and then migrate to spend summer on the feeding grounds with their mother. The exact timing of weaning and separation is variable, but it occurs sometime before, during, or after migration back to the breeding grounds [39, 40]. Past studies of the vocal behavior of immature humpback whales have focused on neonatal calves on the breeding ground [41, 42] and on migration [36, 43, 44]. Recordings of mothers and calves have included both pulsed and tonal call types, and calls were generally relatively short and quiet [41–44]. These quiet calls have been described as acoustic crypsis, and it has been hypothesized that mothers and calves may call at lower amplitudes on the breeding grounds and during migration in order to avoid detection by eavesdroppers such as predators or breeding males [42, 44].

By the time that calves reach the feeding grounds, they are still nursing but begin to exhibit foraging behavior [39, 45, 46]. As the feeding season progresses, calves become more independent, but also more closely follow their mothers’ deeper and longer foraging dives [45, 47]. On the feeding grounds, adults perform solitary and coordinated group foraging behavior and their sound production consist primarily of non-song calls, although song has been recorded on the feeding grounds (e.g., [48–50]). Vocal behavior varies on the feeding ground and may include relatively quiet calling behavior [51], as well as calls specifically associated with certain types of group foraging (e.g., [52]), and a wide diversity of other social call types [53, 54].

It is challenging to study the sound production of young mammals because nursing individuals are usually closely associated with their mother (e.g., [55]) and vocalizations are primarily produced in social contexts, when it is difficult to identify which call is coming from which individual. Biologging tools, such as tags, are instruments with movement and acoustic recording sensors attached to individual animals and can provide valuable fine-scale behavioral data [56]. However, there are often issues with assigning caller identity to sounds recorded on tags when animals are in social groups [57]. Here we leverage simultaneous, synchronous tag data to unambiguously assign caller identity to individuals of known age and sex [58]. From these data, we identify which calls originate from calves and which are produced by adults, allowing the first description of humpback whale calf vocal behavior on a feeding ground. We investigated call amplitude, call timing, and repertoire use in calves and adults to examine how calf vocal behavior differs from that of adults. Since calves are acoustically cryptic through the beginning of their migration [42], we investigated whether there is evidence of continued acoustic crypsis on the feeding ground. To investigate the ontogeny of call production timing, we asked whether calves produce calls in bouts and how the timing of calls in bouts compares between calves and adults. We also looked at how often calls from different individuals in the same group overlap in time. Finally, we classified call types, looked at relative repertoire use across calves and adults, and qualitatively described the calf repertoire. By characterizing the vocal behavior of humpback whale calves relative to adults, we can gain insight into the trajectory of vocal ontogeny in this vocal learning species and the similarities to developmental stages found in other species.

## Methods

### Data collection

Short-term digital acoustic recording tags (Dtag version 2; [59]) were deployed on humpback whales in the Gulf of Maine in the western North Atlantic in the month of July from 2006–2009. Tags were deployed in and around Stellwagen Bank National Marine Sanctuary, a key feeding ground for North Atlantic humpback whales. These archival tags recorded high-resolution sound and movement data and were attached to the back of the whale using suction cups. Dtag hydrophones sampled at a rate of either 64 or 96 kHz.

In addition to tag data, continuous behavioral observations of the tagged individuals were conducted from a small inflatable research vessel. Behavioral data included social affiliations of tagged whales, surface activity, and observable feeding behaviors using an ethogram developed by the Whale Center of New England (e.g., [60–62]). Individual whales were identified in the field by dorsal fin shape and fluke pattern [63]. Calves were classified based on their size, stereotypical behaviors and close, consistent association with a mature female (the mother). Calves were of unknown exact age, but expected to be no more than seven months old at the time they were studied. The sex of calves and demographic data for other tagged whales were provided by the Gulf of Maine Humpback Whale Catalog (Center for Coastal Studies, Provincetown, MA). Whales were classified as male or female based on molecular sex determination [64, 65], a photograph of the genital slit, or, in the case of females, a calving history [66]. Age class was assigned from longitudinal data on the exact or minimum age of each individual. With the exception of the calves, all of the individuals in the study were at least five years old and therefore considered adults [67–69].

### Ethical note

Tagging was conducted under US National Marine Fisheries Service permits 775–185 (to Northeast Fisheries Science Center) and 605–1904 (to the Whale Center of New England) according to all federal and institutional guidelines, and research protocols were approved by the Institutional Animal Care and Use Committees (IACUC) of Duke University, the Pennsylvania State University, and Syracuse University. The tags used here were attached via four suction cups and independently detached from the animal within about 20 hours of attachment, at which point tags float on the surface of the water until the field team recovers them. Tag attachment involves a close approach to the animal in a small rigid hull inflatable boat, where a 7–15 m pole is used to attach the tag to the whale. Individual responses varied from none to short-term (approximately 10 minutes or less) disturbance [70]. Tag placement was also limited to less sensitive areas on the back of the animal between the blowhole and dorsal fin. Individual reactions to tag attachment were monitored and all other behavioral data was collected observationally from a 100–400 m distance.

### Acoustic analysis

#### Focal call assignment

For this study, we used tag data from periods of time when all whales in a group (2–3 individuals) were equipped with tags, when no other non-tagged whales were associated or in close proximity (<500 m) to the group, and when visual observers had recorded focal follow data to confirm the social associations and social context of the tagged whales. Thus, our analysis typically began when the final tag in the group was deployed and ended when the social context changed, one of the tags detached from a whale, or visual observations ended. Because all animals in the group were tagged and no other individuals were in the vicinity, we could compare the relative received level of calls recorded across multiple tags to assign caller ID. We assumed that, regardless of the source level of a vocalization, a sound would have the highest recorded amplitude on the tag attached to the vocalizing whale because this tag would be closest to the sound source [58]. Using signal-to-noise ratio of a vocalization on a single tag may be unreliable [57]; therefore, we chose to leverage synchronous tag data and relative received levels for higher confidence in our caller ID labels.

To label calls, we used a custom script [71] in MATLAB 2019b [72] to visualize synchronous spectrograms and received level plots from each group of tagged whales modified from the Dtag toolbox (animaltags.org). Experienced analysts (VPM and JMZ) visually and aurally browsed the simultaneous data and selected all humpback whale calls. We then labeled calls as focal (i.e., originating from the tagged whale) if they were recorded on only one of the tags in the group or if the call had a higher received level than it did when recorded on another tag in the group. Calls were labeled as nonfocal (i.e., originating from a whale other than the tagged whale) if the received level was lower than it was on another tag. Finally, calls were labeled as indeterminate if the received level was too similar (i.e., less than 1 dB difference) across multiple tags to make a confident assessment, which could indicate calls that originated from more distant whales outside of the tagged group or that animals were very close together. Only calls labeled focal were retained for further analysis. A separate experienced analyst (JMZ, VPM, or DLA) also manually browsed spectrograms from each tag individually in Raven Pro v2.0 [73], and these selections were compared with the MATLAB selections to reduce false positives and false negatives in the dataset. For further details and discussion of this simultaneous tag analysis method, see [58].

#### Signal levels

For all focal calls across all individuals, we calculated a 90% energy window and measured root-mean-squared (RMS) received level (RL) using the *rms* function in MATLAB and converted this value to dB re 1 μPa. This calculation was calibrated for the nominal hydrophone sensitivity of −171 dB re 1 V/μPa [53]. To determine differences in RL between calves and adults, we constructed linear mixed effect models of received level as a function of age class (calf vs adult) with individual ID as a random effect in R using the package *lme4* [74]. We then used AIC values to compare the full model with age class to a null model. All statistical analyses were done in R version 4.1.2 [75].

#### Timing of call production

We looked at the temporal characteristics of calling behavior in calves and adults by conducting a bout analysis and investigating instances of call overlap. We conducted a bout analysis by calculating inter-call intervals (ICI) and estimating a bout end criterion (BEC) to use to assign calls to bouts (see [76, 77]). The BEC is calculated by fitting a “broken-stick” model to a histogram of the logarithm of the inter-call intervals and is a method used widely for investigating bouts in behavioral data [76]. We calculated the ICI for all calls as the time between the onset of one call and the onset of the next call from the same individual. We then log-transformed the data and used the package *diveMove* in R to determine the BEC using the maximum likelihood estimation method [78, 79]. After calculating the BEC, we classified calls with intervals below the BEC threshold as bouts and investigated differences in bout characteristics across calves and adults. We defined bouts as consisting of at least two calls. We measured the duration in seconds of all bouts (from the start of the first call in the bout to the end of the last call in the bout) and the number of calls in all bouts. We calculated the median, interquartile range, and overall coefficient of variation of ICIs for adults and each of the three calves. Finally, we investigated overlap avoidance in communication among tagged whales by looking at how often focal calls overlapped with other focal calls within the same group of animals (e.g., as in [80, 81]).

#### Call classification

**Fig 1:**
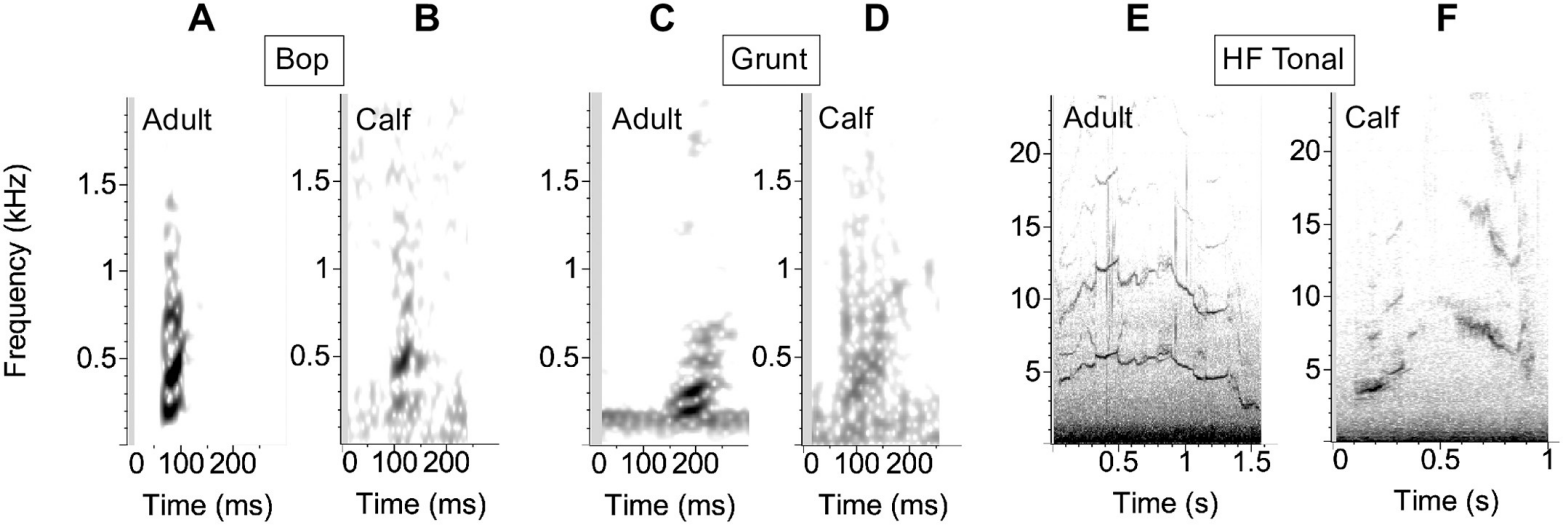
Spectrograms of example calls for different tonal call types produced by adults and calves. A) Adult bop, B) calf bop, C) adult grunt, D) calf grunt, E) adult HF tonal, F) calf HF tonal. The calls were recorded from the following individuals: A) Group 6: mother, B) Group 5: female calf, C) Group 6: adult female, D) Group 6: female calf, E) Group 4: adult female, F) Group 5: female calf. Spectrogram parameters: 4096 DFT, 90% overlap, Hann window.

**Fig 2:**
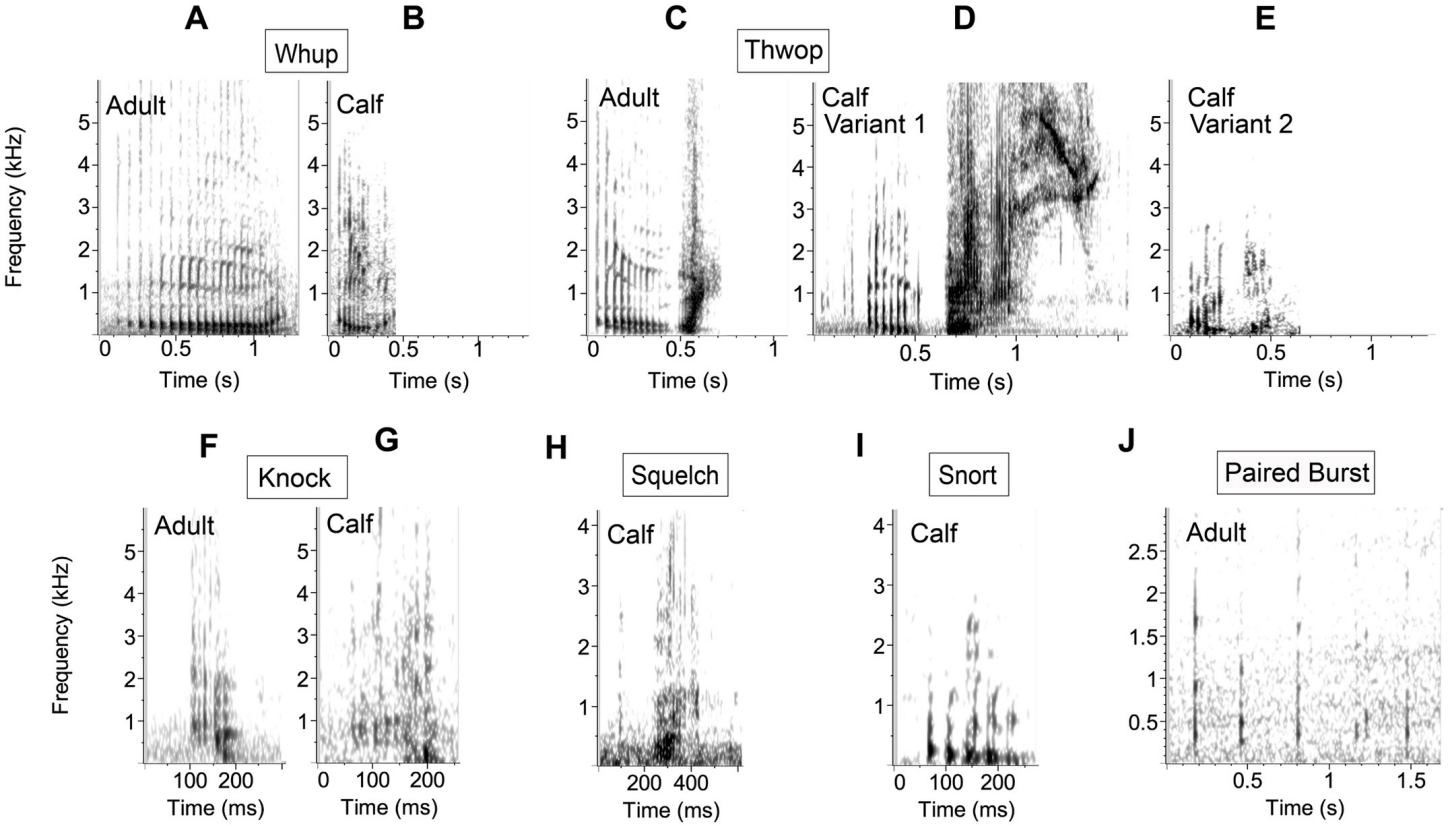
Spectrograms of example calls for different pulsed call types and paired bursts. A) Adult whup, B) calf whup, C) adult thwop, D) calf thwop variant 1, E) calf thwop variant 2, F) adult knock, G) calf knock, H) calf squelch, I) calf snort, J) adult paired burst sequence portion. The calls were recorded from the following individuals: A) Group 6: mother, B) Group 6: female calf, C) Group 6: mother, D) Group 6: female calf, E) Group 6: female calf, F) Group 4: adult female, G) Group 2: male calf, H) Group 6: female calf, I) Group 6: female calf, J) Group 1: adult female 2. Spectrogram parameters: 4096 DFT, 90% overlap, Hann window.

To investigate repertoire use across calves and adults, focal calls were first manually classified into call classes by two experienced analysts (JMZ and DAC) based on established call types in the literature (i.e., [36, 53, 54]). Call classification was done by consensus between the two analysts. We hierarchically classify calls into broad call types and subtypes (similar to [54, 82]). Calls were classified into the following broad call types: high frequency (HF) tonal, low frequency (LF) tonal, low frequency (LF) pulsed, complex, pulse, paired burst, and other. We labeled calls as low or high frequency based on whether the first harmonic was below 2 kHz throughout the call. Tonal calls are narrower in bandwidth than pulsed calls and appear as continuous, frequency-modulated signals on a spectrogram, often with harmonics. Pulsed calls consist of multiple very short, broadband components (pulses) produced in rapid succession to form a single vocalization. Pulsed calls were always low frequency. A singular short, broadband vocalization, similar to one of the individual components of a pulsed call, is referred to here as a pulse. Complex calls are multiple call types combined into one call without a temporal gap, e.g., a pulsed call type and a tonal call type combined continuously. Paired bursts are short pulses or pulsed calls produced in a sequence, as described in [52]. Finally, we classified calls as ‘other’ if they did not fit into these categories (see supplemental material for a call classification decision tree). For LF tonal and LF pulsed, if a call did not fit into one of the subtypes, it was labeled as either LF pulsed (other) or LF tonal (other) as the subtype (Figs 1 and 2). We classified bops and grunts as subtypes within LF tonal calls based on examples in the literature. Within LF pulsed calls, we classified the following subtypes: knock, whup (also referred to elsewhere in the literature as a “wop”), thwop, squelch, and snort (Figs 1 and 2). We classified thwop-like sounds from calves as thwop variant 1 and thwop variant 2, where the first variant is similar to an adult thwop with additional components at the end and variant 2 is a shorter, simpler version of a thwop (Fig 2D and 2E). We then compared repertoire use across individuals of different ages by comparing adult and calf proportional call type use during the analysis period. Repertoire use was calculated as the total number of times a call type was produced by either calves or adults divided by the total number of calls produced by either calves or adults. For some call types, some calf calls had clear structural or spectral differences from the standard adult calls despite fitting into the same call type classification. These calf variants of standard call types were noted and are qualitatively described.

## Results

In total, we browsed 46 hours and 52 minutes of tag acoustic data across 16 different individuals and 7 different groups (3 groups with calves and 4 groups only containing adults) for periods when visual observations confirmed that all individuals in a group were tagged, and non-tagged individuals were not in close proximity to the group. Our data included 13 individuals for whom calls were detected during the analysis period. We detected 982 focal calls across all tags, with some individuals producing few or no calls and others producing over 300 calls (Table 1). Group 6 alone produced more than 75% (>750 calls) of the total focal calls detected.

**Table 1:** Summary of tag data.

Date	Group	Analysis duration (hh:mm)	Whale class	Total number of focal calls
July 19, 2006	1	1:28	Adult Female 1	0
			Adult Female 2	20
July 17, 2007	2	2:40	Male Calf	3
			Mother	0
July 7, 2008	3	2:27	Adult Male	8
			Adult Female	13
July 14, 2008	4	0:31	Adult Female	44
			Adult Male	11
July 22, 2009	5	3:47	Female Calf	15
			Mother	19
			Adult Female	78
July 20, 2009	6	6:55	Adult Female	330
			Female Calf	302
			Mother	133
July 29, 2009	7	0:17	Adult Female	0
			Adult Male	6

The table lists exact date of tag deployment, group number, analysis duration for each individual, individual whale class (based on age, sex, and role), and total number of focal calls for each tagged individual. Analysis duration was determined as the period when all whales in the group were tagged and no non-tagged whales were in the vicinity.

### Signal levels

Calves on the feeding ground did not show acoustic crypsis. All three calves produced calls during the recording period. The average received level of calf calls on the tags was 143 dB re 1 μPa (+/- 8 dB re 1 μPa SD) and the average received level of adult calls was 141 dB re 1 μPa (+/- 13 dB re 1 μPa S.D.). The received level of adult calls was more variable (higher standard deviation) than that of calves. The AIC value of the null model was lower than that of the full model, indicating that age class is not a significant predictor of received level (ΔAIC = 3.9).

### Timing of call production

Both calves and adults produced calls in bouts based on a predicted BEC of 2.2 s (i.e., all calls with an inter-call interval of less than 2.2 s were considered part of a bout). The calf in Group 2 produced 1 bout, the calf in Group 5 produced 4 bouts, and the calf in Group 6 produced 40 bouts. Because of the skewed call rate across the three calves, we report results for each individual calf separately. Although the data are dominated by the behavior of the calf in Group 6, overall the calf bouts had longer median ICIs than adult bouts (Group 2 Calf ICI = 0.62 s, Group 5 Calf median ICI = 0.62 s, Group 6 Calf median ICI = 0.59 s, Adult median ICI = 0.47 s). The inter-quartile range was higher for bout ICIs from calves (IQR: Group 5 Calf = 0.45, Group 6 Calf = 0.77) than those from adults (IQR = 0.39). Bouts from calves were also shorter in duration (Group 2 Calf bout duration = 0.86 s, Group 5 Calf median bout duration = 1.2 s, Group 6 Calf median bout duration = 1.6 s) than adult bouts (median bout duration = 2.1 s).

We detected 10 instances of temporally overlapping calls across all tags in the dataset, all occurring in groups with calves. No calls overlapped with each other in time in any of the groups with only adults. Of the 10 instances where there was temporal overlap, 8 involved a call from one of the calves.

### Call repertoire

**Fig 3:**
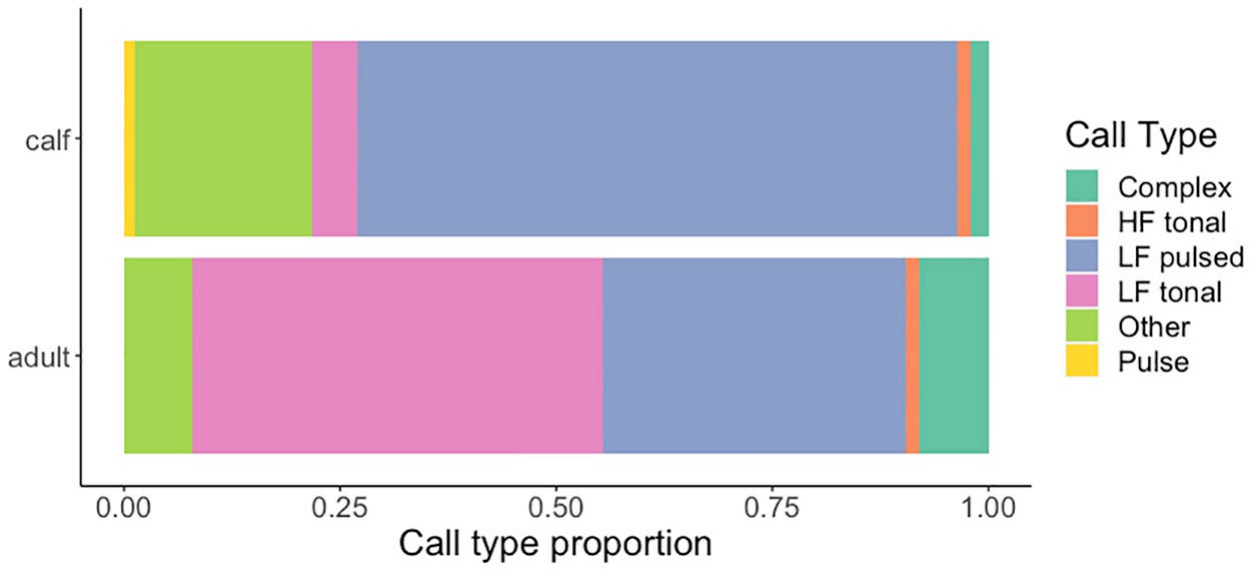
Broad call types recorded from the mother (adult) and calf in Group 6 represented as proportions of total recorded repertoire for that individual. For example, LF pulsed sounds made up about 80% of all calls produced by the calf. Broad call types shown are Complex, HF tonal, LF pulsed, LF tonal, Other, Paired Burst, and Pulse.

Although there were differences in the relative proportional use of different call types and subtypes, overall calf and adult repertoire diversity was very similar (e.g., Fig 3). Calves and adults produced all broad call types except paired burst sounds, which were only detected from adult whales. The Group 2 calf produced 2 knocks and 1 LF pulsed call. The Group 5 calf produced mostly HF tonal calls (13 total calls), and the rest were LF tonal (6 total calls, 4 bops and 2 other LF tonal calls). On a broad call type scale, adults produced proportionally more LF-tonal sound types (including bops, grunts, and other low-frequency tonal calls). In terms of specific call subtypes, 51% of the calls recorded from adults were bops, whups, and grunts (Table 2). Knocks, HF tonal calls, snorts, squelches, and other LF-pulsed sounds made up the majority of calls recorded from each of the three calves (Table 2). Thwop variants were not recorded from any adults (Table 2). To control for call rate and behavioral context, we also compared the broad call type proportional use of the mother and her calf in Group 6 who produced a similar number of total calls during the analysis period (Fig 3). The calf produced all of the call types that the mother produced, but the mother produced more LF tonal calls and the calf produced more LF pulsed and other call types.

**Table 2:** Proportional use of specific call subtypes for adults and calves.

Call type	Subtype	Adult proportional use	Group 2 Calf proportional use	Group 5 Calf proportional use	Group 6 Calf proportional use
LF tonal	Bop	0.286	0	0.211	0.003
LF pulsed	Whup	0.127	0	0	0.009
LF tonal	Grunt	0.116	0	0	0.015
LF pulsed	Knock	0.092	0.667	0	0.076
HF tonal		0.079	0	0.684	0.015
Paired burst		0.079	0	0	0
LF pulsed	LF pulsed	0.056	0.333	0	0.218
Complex		0.054	0	0	0.003
LF pulsed	Snort	0.022	0	0	0.170
LF tonal	LF tonal	0.022	0	0.105	0.033
LF pulsed	Thwop	0.016	0	0	0.109
Other		0.014	0	0	0.009
LF pulsed	Squish	0.013	0	0	0.109
Pulse		0.011	0	0	0.009
LF pulsed	Squelch	0.01	0	0	0.139
LF pulsed	Pseudo-thwop	0.003	0	0	0.048

Rows are sorted from highest to lowest adult proportional use. The proportions in each column add to 1. The top 3 calls that make up 50% of the repertoire for adults and for each calf are highlighted in gray.

Finally, there were a few examples of call types that appeared stereotyped among adults, but one of the calves produced variants of these stereotyped calls with structural differences. We classified these calls as thwop variants 1 and 2. The female calf from Group 5 produced multiple calls which are clearly thwops based on their initial structure but which include additional tonal and pulsed components at the end, and we classified these as thwop variant 1 (Fig 2C and 2D). This same calf produced other thwop-like sounds, which we classified as thwop variant 2, that have the same two-component structure as adults (i.e., a downswept pulsed sound followed by an upswept pulsed sound), but which are shorter, simpler, and have a more pulsed and less pronounced upsweep than the adult thwop. These variants were included as thwops rather than a distinct call type because of the stereotyped structure and because these thwop variants were never recorded from adults in the dataset. We also found some other call variants in the dataset. There were very few calf whups recorded on the tags, but those that were recorded and classified as whups, as well as some of the other LF pulsed sounds, seemed to be whup variants or a variant somewhere in between a whup and a thwop (Fig 2A and 2B). These whups and LF pulsed sounds from calves primarily varied in the structure of the frequency descent or ascent of the call. These calf calls contrast adult whups, which were very stereotyped and primarily varied in duration. Calves often repeated thwop variant 2 and other LF pulsed sounds in single call type bouts.

## Discussion

Using synchronous tag data from groups of whales, we could identify calls produced by different individuals in small groups of humpback whales using relative amplitude comparisons across recordings [58]. This method allowed us to investigate acoustic behavior on an individual level as it relates to age class, which was not possible in the past. We described calls recorded from 3 dependent calves on the feeding ground including call amplitude, call timing, and repertoire use compared to adults.

Age class (calf vs adult) was not a significant predictor of call received level, which contrasts with evidence of acoustic crypsis in humpback whale calves on the breeding ground [42] and on migration [44]. It has been hypothesized that calves may call quietly in those contexts in order to avoid detection by predators or breeding males [42, 44]. There may be less risk of predation for calves on the feeding ground, either because there are fewer interactions with potential predators [83] or because the calves have grown in size and are less at risk. Calf call amplitude could also increase in tandem with anatomical growth [8, 84].

Both calves and adults produced calls in bouts with inter-call intervals less than 2.2 s, similar to previously reported durations for the inter-call interval of humpback whale call bouts [77]. Calf bouts had longer median ICIs than adult bouts, which could relate to the ontogeny of rhythm and timing of vocal production. The inter-quartile range of the ICIs in bouts from calves were higher than in adults, indicating more variability in the timing of calls within calf bouts. This result is consistent with other studies of the ontogeny of vocal timing in other taxa, including humans and birds, where younger individuals exhibit less precision in the timing of their vocalizations [13–15]. Calf bouts were also shorter in duration and number of calls than adult bouts. Bout durations and number of calls per bout have been shown to increase from calves to adults in other species as well, and bout duration could be an indication of stamina or less complex vocal behavior (spotted hyenas, *Crocuta crocuta*: [85]; North Atlantic right whales, *Eubalaena glacialis*: [9]). Future studies can also investigate the role that different call types play in bout timing across individuals. Being able to produce acoustic sequences, like call bouts, also has important implications for song learning for male humpback whales.

We also found preliminary evidence of overlap avoidance in humpback whale vocal exchanges based on the lack of temporally overlapping calls in the dataset. The only exceptions to this overlap avoidance occurred in groups with calves, where there were 10 instances of calls that overlapped with each other in time. Although we could not robustly test the probability of call overlap using this dataset, these preliminary results suggest future research into call timing in humpback whale vocal exchanges. Overlap avoidance is a fundamental feature of turn-taking [80, 81, 86]; however, it is also learned during ontogeny in birds and mammals [13–15]. In fact, human and other primate infants also show higher levels of overlapping vocalizations early in life, decreasing with age [13, 14]. These results related to vocal timing, both within sequences from a single individual as well as in vocal exchanges, are particularly interesting because studies of such features are only possible with robust caller identification methods [87]. Call overlap did occur in the two groups with three individuals, so it is possible that large groups of whales simply show more call overlap. Future research should investigate the effects of group size and age composition on the number of call overlaps to test whether overlap avoidance is actually influenced by age.

At a broad call type level, calves made almost all of the call types that adults did but used different call types at different rates. Paired bursts were the only call type produced by adults but not by calves. Group 6, a group with two adults and one calf, and Group 2, a mother/calf pair, did not exhibit any paired bursts. Paired bursts are associated with coordinated bottom feeding [52], a strategy these groups likely did not use during the analysis period. In Group 5, another group with two adults and one calf, only one of the adults produced paired bursts. Calves may be unable to produce paired bursts by six months of age or may not have participated in the bottom feeding behavior. All other call types were recorded from both age groups, indicating that humpback whale calves can produce most, if not all, of the adult repertoire by about six months of age. Calves and adults produced the rest of the calls they had in common in different proportions. Calves used LF pulsed and HF tonal calls most often, while adults used LF tonal calls most often, which could represent a shift in repertoire use with age. However, without being able to control for contextual differences across groups, it is hard to rule out their potential contributions to these observed differences in repertoire use. Bops were produced by both calves and adults, as were grunts.

For some specific call subtypes, we observed from calves some structural variants of stereotyped adult calls like thwops and whups. The thwop variants were not produced by any of the adults, do not resemble other described call types in the literature, and were often produced in bouts. These observations resemble protosyllables and correspond to the definitions of babbling described in other species [6, 27–30]. These behaviors may represent vocal practice, exploration, and sensorimotor learning, and future research should work to explore additional evidence that these developmental stages are present in humpback whale vocal ontogeny.

Manual call classification is subjective, and it is possible that some call types, such as the calf thwop variants, are functionally distinct. It is also possible that some of the calls we found produced only by calves or primarily by calves (some of the other LF pulsed calls and squelches) are also calf variants of and functionally comparable to stereotyped adult calls and thus should be included in other call type categories instead of separated. The call variants described here come from one calf, likely because we recorded very few calls from the other two calves in this dataset. While these examples may represent the behavior of one individual rather than a trend in vocal behavior across calves in general, these qualitative descriptions lay a useful groundwork for further investigation in the future with a larger sample size. We also only have data from a short snapshot of the calf’s behavior. Although we cannot characterize calf behavior in general from these anecdotes, the data do show what these individuals are capable of. Additional recordings that can be attributed to immature humpback whales will allow for further explicit comparisons between the repertoire of adults and calves/juveniles to differentiate adult calls from adult-like calls and protosyllables. The lack of a complete vocal repertoire catalogue for adult humpback whales on the feeding ground prevents us from determining whether adults ever produce any of the recorded calf vocalizations. The vocal repertoire of adult humpback whales is also graded [54, 88, 89], so it is challenging to differentiate between variation that is standard in the adult repertoire and variation that may result from ontogenetic processes.

It is important to note that this caller identification method involves multiple tag deployments on specific individuals, making it challenging to build a large dataset for answering these types of questions about individual acoustic behavior. The dataset we describe here varies in the duration of data analyzed for each group and in the call rate of each individual. The variability in call rate led to an overrepresentation in the data of a few more vocally active individuals, which may bias the results. Future studies should work to replicate these analyses to verify whether these results hold true across a larger sample size. Although the dataset here consists of only three calves, with one calf producing the majority of calls, these results are still a first look at the behavior of calves on the feeding ground, when they are still dependent on their mother, and provide valuable insight into the vocal ontogeny of humpback whales.

## Conclusion

We provide the first description of the acoustic behavior of humpback whale calves on the feeding ground using synchronous tag data to assign caller identity based on relative amplitude differences of calls recorded on multiple tags. We found that calves are not acoustically cryptic on their feeding grounds as they appear to be on the breeding grounds and during migration. Both calves and adults produce calls in bouts, but calf bouts are shorter and have more variable inter-call intervals than those of adults. Along with evidence of temporal call overlaps only occurring in groups with calves, these data suggest that call timing may also develop during ontogeny. Calves can produce most of the adult repertoire, but use different call types in different proportions. Finally, we described variants of adult call types produced by calves as well as some calls produced only by calves that may be examples of protosyllables and babbling, as described in other vocal learning species, including humans.

## Supporting information

S1 Fig A flowchart visualizing the call classification process and example spectrograms of the call types. Blue text represents broad call types and red text represents call subtypes.
(TIF)

S1 File Selection tables including call classifications, inter-call interval measurements, and received level measurements. (CSV)

S2 File Sound clips corresponding to spectrograms of adult and calf examples of each call type listed in S1 Fig, Figs 1 and 2. (ZIP)
