# Supplementary figures and images for: Acoustic behavior of humpback whale calves on the feeding ground: Comparisons across age and implications for vocal development
Source: PLoS One. 2024 May 29;19(5):e0303741. doi: 10.1371/journal.pone.0303741 (PMC11135678; doi:10.1371/journal.pone.0303741)

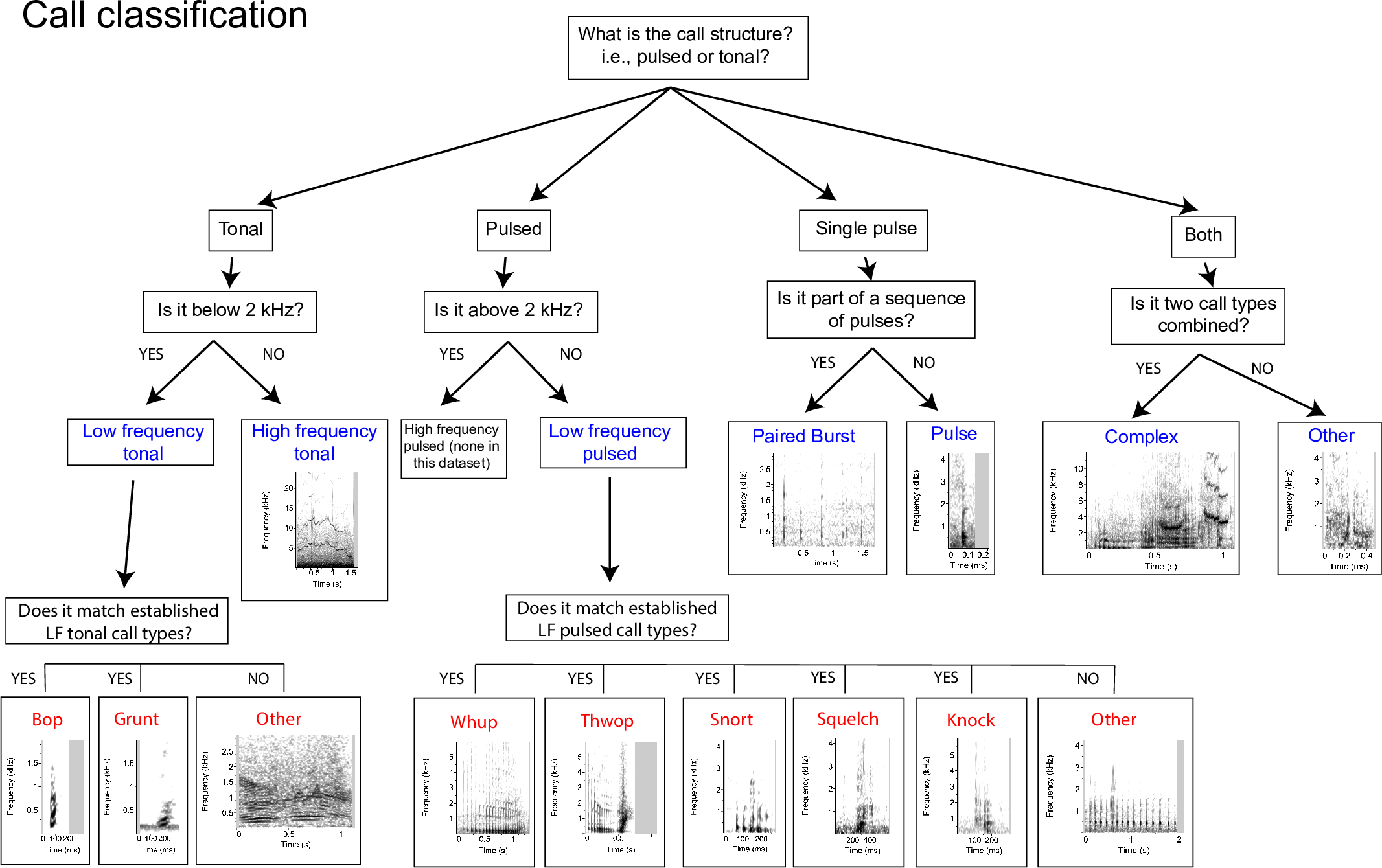

Supplement: S1 Fig — Blue text represents broad call types and red text represents call subtypes. (TIF) [file pone.0303741.s001.tif]
